# Revision of Energy Metabolism Adaptations in High-Level Athletes: From Physical Performance Enhancement to Potential Therapeutic Targets in Mental Disorders

**DOI:** 10.3390/cimb48050498

**Published:** 2026-05-11

**Authors:** Ane Larrea, Mariyem Naji, Marina Gulak, Maria Torrecilla, Gabriel Barreda-Gómez

**Affiliations:** 1 Department of Pharmacology, Faculty of Medicine and Nursing, University of the Basque Country, UPV/EHU, 48940 Leioa, Spain; anelarrea96@gmail.com (A.L.); maria.torrecilla@ehu.eus (M.T.); 2 Faculty of Experimental Sciences, University of Huelva, 21071 Huelva, Spain; mariyemnaji30@gmail.com; 3 Urduliz Alfredo Espinosa Hospital—OSI Uribe, 48610 Urduliz, Spain; marina-gulak@libero.it; 4 Research and Development Department, Buscalud Biofarma S.L., 48003 Bilbao, Spain

**Keywords:** mitochondria, metabolic pathways, athletes, mental health

## Abstract

High-level athletic performance requires the implementation of personalized strategies based on the analysis of metabolic pathways involved in energy production: phosphagen, glycolytic, and oxidative pathways. In this context, mitochondria play an essential role as the central regulator of energy production, being closely linked to these three pathways. Exercise boosts cellular respiration, which can also be optimized by nutritional interventions and targeted supplementation, promoting mitochondrial biogenesis, reducing oxidative stress and increasing ATP production. These metabolic adaptations improve athletic performance, accelerate recovery processes, and reduce the risk of injury, adapting to the physiological characteristics of each athlete. Moreover, some of these metabolic adaptations converge on specific targets whose expression or activity is also altered in mental disorders. Therefore, the aim of this review is to analyze mitochondrial adaptations induced by exercise and supplementation, evaluating their impact on the phosphagen, glycolytic, and oxidative metabolic pathways, as well as their relationship with optimizing performance and recovery in high-level athletes, with special attention to their potential application to mental health.

## 1. Introduction

High-level athletic performance depends on the organism’s ability to generate and utilize energy efficiently and sustainably, which varies according to the intensity, duration, and modality of exercise. Cellular energy is primarily obtained through three main metabolic pathways: the phosphagen system (ATP-PCr), anaerobic glycolysis, and aerobic oxidative metabolism. Each pathway has specific biochemical characteristics and limitations [1]. The phosphagen system is activated during short, explosive efforts lasting less than ten seconds and allows for the rapid regeneration of ATP from phosphocreatine without requiring oxygen [2]. Anaerobic glycolysis predominates during moderate- to-high-intensity exercise of intermediate duration, approximately between ten seconds and two minutes, generating ATP via glycolysis with lactate as a byproduct [3]. Finally, the oxidative pathway sustains prolonged efforts of more than two minutes through the oxidation of carbohydrates, lipids, and, to a lesser extent, amino acids [1].

Within this energetic framework, mitochondria act as a central hub by integrating the different pathways and optimizing energy production according to demand, thereby facilitating the transition between systems. Their functional capacity determines ATP production efficiency and fatigue tolerance, both of which are critical for athletic performance [4]. Although the phosphagen system does not directly rely on mitochondrial respiration, mitochondria contribute decisively to phosphocreatine resynthesis during post-exercise recovery through ATP production, which is essential for maintaining explosiveness in repeated efforts [5]. The lactate generated in the cytosol in anaerobic glycolysis can be transported into the mitochondria, where it is converted to pyruvate and subsequently oxidized in the Krebs cycle. This “lactate–pyruvate shuttle” improves energy efficiency and limits the accumulation of metabolites associated with muscle fatigue [6]. The oxidative pathway, in turn, depends almost entirely on mitochondria. Mitochondrial oxidative phosphorylation, coupled to the electron transport chain (ECT) and proton gradient, enables a high yield of ATP from carbohydrates and fats. It is estimated that more than 90–95% of ATP generated under aerobic conditions derives from this mitochondrial process, while the remaining fraction corresponds to cytosolic glycolysis, which supplies pyruvate for subsequent oxidation [7]. Thus, mitochondrial density and efficiency condition both maximal oxygen consumption (VO_2_max) and tolerance to prolonged exertion [8].

Physical exercise induces structural and functional adaptations in mitochondrial biology. Among these, mitochondrial biogenesis stands out, as it increases mitochondrial number and size through the activation of regulators such as peroxisome proliferator-activated receptor gamma coactivator 1-alpha (PGC-1α), nuclear respiratory factor (NRF1/2), and mitochondrial transcription factor A (TFAM), thereby enhancing oxidative capacity and muscle endurance [9]. Improvements are also observed in respiratory chain efficiency, with increased activity of complexes I–IV and ATP synthase, reducing uncoupling and oxidative stress [10]. Selective mitophagy, mediated by PTEN-induced kinase 1 (PINK1) and PARKIN, ensures the removal of dysfunctional mitochondria and the maintenance of a healthy mitochondrial pool [11]. In addition, exercise augments the activity of oxidative enzymes involved in the Krebs cycle, β-oxidation, and electron transport, thereby promoting greater ATP production per unit of substrate consumed [12]. These adaptations, however, are dependent on the type of training: endurance exercise promotes greater mitochondrial density and efficiency, while strength training generates more localized and fiber type II-specific modifications [13].

Nutrition and supplementation also play a key role in modulating mitochondrial function. Various antioxidant compounds mitigate damage caused by reactive oxygen species (ROS) generated during intense exercise [14]. Other dietary-derived compounds promote both mitophagy and biogenesis, leading to improved energy efficiency and muscle recovery [15]. Furthermore, nutrients including branched-chain amino acids (BCAAs), creatine, and omega-3 fatty acids contribute to mitochondrial signaling and the preservation of inner membrane integrity [16]. Together, these strategies synergize with physical training, enhancing performance, accelerating recovery, and reducing the risk of injury.

The influence of mitochondria extends beyond athletic performance, impacting recovery, injury prevention, and mental health. Their efficiency regulates metabolite clearance, controls inflammatory processes, and ensures energetic homeostasis [17]. Optimal mitochondrial function therefore facilitates post-exercise recovery by reducing fatigue, limiting lactate accumulation, and decreasing oxidative stress [18]. It also contributes to injury prevention by maintaining muscle integrity and reducing overload on type II fibers. At the cerebral level, mitochondria play a fundamental role in regulating neuroplasticity, energy metabolism, and antioxidant signaling, suggesting that their optimization may improve cognition and reduce the risk of neuropsychiatric disorders [19]. However, prolonged exposure to intense exercise, strict dieting, and extreme stress—common in professional athletes such as football players, sumo wrestlers or gymnasts, among others—can significantly increase the risk of both physical and mental disorders such as anxiety, depression, anorexia, bulimia or neurodegenerative diseases.

Accordingly, the aim of this review is to analyze mitochondrial adaptations induced by exercise and supplementation, evaluating their impact on the phosphagen, glycolytic, and oxidative metabolic pathways, as well as their relationship not only with optimizing performance and recovery in high-level athletes but also with mental health.

## 2. Methodology

This work constitutes a narrative review, as the primary objective is to synthesize and integrate evidence across multiple biological domains—exercise physiology, mitochondrial biochemistry, nutritional science, and neuropsychiatry—rather than to quantify pooled effect sizes. A systematic literature search was conducted in PubMed/MEDLINE, Scopus, and Web of Science, with no language restrictions applied. The search was structured around three thematic axes related to high-level athletes: (i) mitochondrial adaptations to exercise; (ii) nutritional modulators of mitochondrial function; and (iii) mitochondrial dysfunction in psychiatric, metabolic, and neurodegenerative disorders.

Articles were included if they met the following criteria: (i) original research articles, systematic reviews, meta-analyses, or narrative reviews published in peer-reviewed journals; (ii) studies reporting outcomes related to mitochondrial structure, function, biogenesis, dynamics, or redox regulation; and (iii) human studies, animal models, or in vitro studies providing mechanistic insights relevant to the review objectives. Articles were excluded if they (i) were published only as conference abstracts without full-text availability; (ii) reported exclusively on non-mammalian organisms with limited translational relevance; or (iii) lacked sufficient methodological detail to assess data quality. Reference lists of retrieved articles were additionally screened to identify relevant publications not captured by the database searches. Final source selection was guided by relevance to the review’s objectives, methodological quality, and publication recency, prioritizing human studies and, where available, randomized controlled trials over observational or mechanistic evidence alone.

## 3. Energy Systems and Mitochondrial Interactions

### 3.1. Phosphagen Pathway

**Figure 1:**
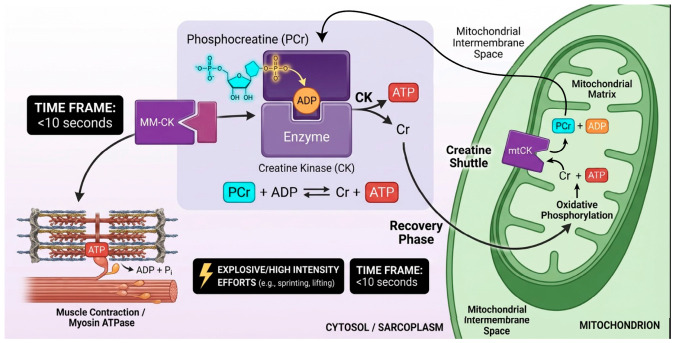
**Phosphagen (ATP-PCr) pathway: dual-phase energy coupling in high-intensity exercise.** The diagram illustrates the two operational phases of the phosphagen system. During exercise (sarcoplasmic phase), creatine kinase (CK) catalyzes the near-instantaneous transfer of a phosphate group from phosphocreatine (PCr) to ADP, sustaining myosin ATPase activity independently of oxygen. During recovery (mitochondrial phase), the mitochondrial CK isoform (mtCK) uses oxidative phosphorylation-derived ATP to resynthesize PCr via the creatine shuttle, thereby restoring the cell’s high-energy phosphate reserve. The bidirectional arrow highlights the reversibility of the CK reaction and its role as a metabolic buffer linking mitochondrial output to cytosolic energy demand.

The phosphagen (ATP–PCr) system is the fastest mechanism for ATP regeneration in skeletal muscle, supporting high-intensity, short-duration efforts (<10 s) such as sprints or jumps, where energy demand exceeds glycolytic and oxidative capacity (Figure 1).

Phosphocreatine (PCr) serves as an immediate energy reservoir, donating a phosphate group to ADP via creatine kinase (CK) [20]. This reversible reaction adapts to energy demands: during exercise it produces ATP, while during recovery it uses mitochondrial ATP to restore PCr [21]. CK isoforms are strategically localized: cytosolic CK supports ATP use at contractile sites, while mitochondrial CK (mtCK) enables PCr resynthesis through the creatine shuttle, linking mitochondrial ATP production to cytosolic energy needs [22,23]. This system operates independently of oxygen and produces minimal metabolic byproducts, maintaining pH balance during initial effort [22]. However, limited PCr stores (20–30 mmol·kg^−1^) restrict its contribution to ~6–10 s of maximal activity, after which glycolysis predominates [24]. PCr resynthesis depends on mitochondrial oxidative phosphorylation, driven by ADP accumulation and supported by substrate oxidation and nucleotide transport via ANT [25,26].

Training induces key adaptations: high-intensity interval training (HIIT) increases mitochondrial density, respiratory efficiency, and PCr resynthesis rates [27], while upregulating CK and mtCK expression [18]. Endurance training also enhances oxidative capacity, indirectly supporting PCr recovery. Additional adaptations include improved antioxidant defenses and lactate utilization, supporting mitochondrial function and PCr regeneration [28]. These changes improve performance by accelerating PCr recovery (within 2–5 min), enabling repeated high-power efforts and delaying fatigue. Training strategies that combine intense bouts with active recovery further optimize the interaction between anaerobic and aerobic systems [24,29].

### 3.2. Glycolytic Pathway

**Figure 2:**
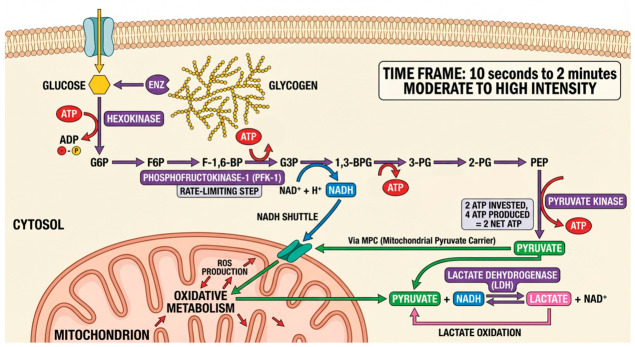
**Glycolytic pathway: cytosolic ATP production and metabolic fate of pyruvate.** The figure maps the sequential enzymatic breakdown of glucose and glycogen to pyruvate, with PFK-1 as the key allosteric control point. The net energetic yield (2 ATP per glucose after initial investment) and the two divergent fates of pyruvate are highlighted: (i) reduction to lactate by LDH under high glycolytic flux, enabling NAD^+^ regeneration and sustained anaerobic ATP production; and (ii) mitochondrial entry via the pyruvate carrier (MPC) for aerobic oxidation when oxygen supply permits.

The glycolytic pathway is a key energy system for moderate- to high-intensity efforts lasting ~10 s to 2 min, when phosphagen stores decline and oxidative metabolism is not yet maximal [30]. It produces ATP anaerobically by converting glucose or glycogen into pyruvate through regulated enzymatic steps (Figure 2). Key control enzymes include hexokinase, PFK-1 (rate-limiting, regulated by cellular energy status), and pyruvate kinase, which adjusts ATP production to demand [31,32,33].

Although primarily anaerobic, glycolysis is closely linked to mitochondrial function. Pyruvate can enter mitochondria via the mitochondrial pyruvate carrier for oxidation, while lactate produced during intense exercise can be reused as a substrate after reconversion to pyruvate [34,35]. This lactate shuttle helps maintain NAD^+^/NADH balance and prevents inhibition of glycolysis due to acidification, sustaining ATP production during high-intensity efforts. Mitochondria also regulate oxidative stress associated with glycolysis. Increased electron transport activity elevates ROS production, which at moderate levels promotes mitochondrial biogenesis via PGC-1α, NRF1/2, and TFAM, enhancing oxidative capacity and ATP efficiency [36,37]. However, excessive ROS can impair key metabolic enzymes, requiring antioxidant defenses such as SOD, catalase, glutathione, and vitamins C and E to preserve function [38,39].

Training enhances glycolysis–mitochondria integration by increasing mitochondrial density, enzyme activity (e.g., PDH, PFK), and metabolite transport, as well as antioxidant capacity [40]. These adaptations improve pyruvate and lactate oxidation, maintain NAD^+^ levels, and limit ROS damage, allowing for better performance, faster recovery, and reduced metabolite accumulation during repeated high-intensity exercise [41].

### 3.3. Oxidative Pathway

**Figure 3:**
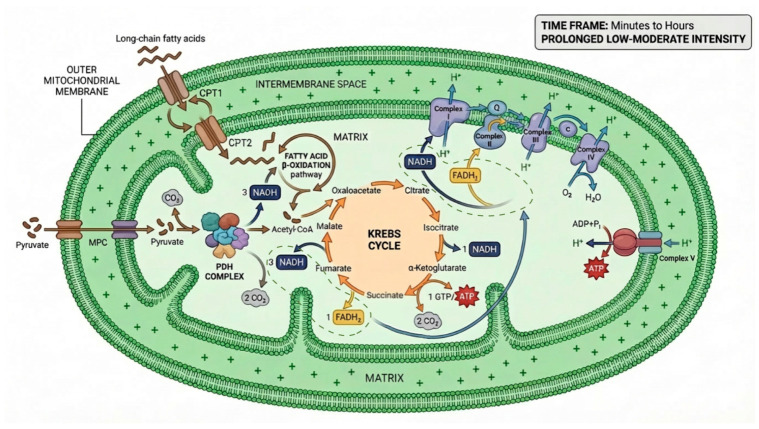
**Oxidative pathway: substrate integration and chemiosmotic ATP synthesis.** The diagram summarizes the three sequential stages of aerobic energy transduction. Substrate entry: long-chain fatty acids undergo β-oxidation after mitochondrial import via CPT1/CPT2; glucose-derived pyruvate is converted to acetyl-CoA by PDH. Krebs cycle: acetyl-CoA oxidation generates NADH and FADH_2_, the primary electron donors. Electron transport chain (ETC): electrons flow through complexes I–IV, driving proton translocation across the inner mitochondrial membrane; the resulting electrochemical gradient powers ATP synthase (complex V), with O_2_ as the terminal electron acceptor.

The oxidative pathway is the main energy system during prolonged, low- to moderate-intensity exercise, sustaining ATP production for minutes to hours [42]. It utilizes carbohydrates, fatty acids, and amino acids, and depends on oxygen availability, mitochondrial function, and antioxidant capacity (Figure 3) [43]. It also supports recovery by regenerating PCr, oxidizing lactate, and restoring ATP levels [44]. Biochemically, glycolysis produces pyruvate, which enters mitochondria via the MPC and is converted to acetyl-CoA by PDH, generating NADH [45]. Acetyl-CoA enters the Krebs cycle, producing reducing equivalents (NADH, FADH_2_) for the electron transport chain (ECT). Fatty acids contribute via β-oxidation, and amino acids can replenish Krebs cycle intermediates during prolonged exercise [46,47].

Regulation depends on energy and redox status: PDH and Krebs cycle enzymes are controlled by ATP, ADP, NADH, and related metabolites [47,48]. The ECT generates a proton gradient that drives ATP synthesis, with efficiency reflected by the P/O ratio, a key determinant of endurance performance [45,49]. Training induces major adaptations, including increased mitochondrial density, enzyme activity, and oxidative capacity, improving substrate utilization and ATP production efficiency [50]. Both endurance and high-intensity training enhance lactate oxidation, PCr recovery, and integration with glycolysis, supporting sustained and intermittent performance [36].

The oxidative pathway works dynamically with anaerobic systems: during intermittent exercise, phosphagen and glycolysis provide immediate ATP, while oxidative metabolism maintains energy supply, oxidizes lactate, and delays fatigue [51]. Its efficiency depends on oxygen transport. Hemoglobin delivers O_2_, with release enhanced by 2,3-BPG and the Bohr effect, improving oxygen unloading in active muscle [52,53,54]. Oxygen diffusion follows Fick’s law, with capillary density and microvascular flow determining delivery [55]. Myoglobin facilitates intracellular O_2_ transport and storage, maintaining mitochondrial oxygen availability [56,57]. Together, oxygen delivery and extraction (a–vO_2_ difference) determine VO_2_max and the capacity to sustain oxidative metabolism under high demand [58].

## 4. Nutritional Modulators of Mitochondrial Function

A comprehensive summary of these compounds, including their level of evidence, effect size, and key limitations, is presented in Supplementary Materials Table S1.

### 4.1. Creatine

Creatine, widely used in sports, exerts effects on mitochondrial function beyond ATP resynthesis via the ATP–PCr system [59] (Table S1). It promotes mitochondrial biogenesis by upregulating transcription factors such as NRF-1, NRF-2, and TFAM (25–35% increase) [60,61], likely through AMPK activation and subsequent PGC-1α signaling [62]. Creatine also enhances mitochondrial respiration sensitivity to ADP, improving oxidative phosphorylation efficiency and ATP production during exercise [63,64]. Animal and human studies show increased ADP-dependent respiration, VO_2_max, and energy efficiency [65,66], supported by mtCK activity, which optimizes phosphate transport and reduces ROS generation [67]. However, improvements in VO_2_max attributed to creatine in some studies are modest and not consistently replicated across trials, particularly in already well-trained athletes, where the ergogenic ceiling may be lower [65]. Additionally, creatine contributes to redox homeostasis by maintaining NAD^+^ levels, supporting NADH production, and preserving NADPH for glutathione (GSH) regeneration [59]. It enhances antioxidant defenses, reduces oxidative damage, and may stimulate pathways such as the pentose phosphate pathway, improving recovery and delaying fatigue [68]. Nonetheless, the antioxidant evidence remains largely mechanistic, and controlled trials specifically measuring creatine’s redox effects independent of training are scarce [59,68]. Overall, creatine supports mitochondrial biogenesis, respiration, and antioxidant capacity, enhancing performance and recovery [59], although the magnitude and consistency of these effects across populations warrant cautious interpretation.

### 4.2. NAD^+^ Precursors

Nicotinamide adenine dinucleotide in its oxidized form (NAD^+^) acts as a central integrator. NAD^+^ is a key regulator of cellular bioenergetics and redox balance [69]. Supplementation with precursors such as nicotinamide riboside (NR) and nicotinamide mononucleotide (NMN) increases NAD^+^ via the salvage pathway, enhancing mitochondrial electron transport and reducing reductive stress during exercise [70,71]. Elevated NAD^+^ activates SIRT1, which enhances PGC-1α activity and promotes mitochondrial gene expression and biogenesis [72]. In mitochondria, SIRT3 improves enzyme efficiency and reduces ROS production [73]. These adaptations are associated with improved VO_2_max, oxidative capacity, and mitophagy via PINK1/Parkin pathways [74,75]. However, the translational value of these findings must be qualified. The most robust evidence for NAD^+^ precursor efficacy comes from rodent models and from studies in older or metabolically compromised individuals, in whom baseline NAD^+^ levels are substantially depleted [69]. In healthy, trained populations, where NAD^+^ metabolism is already upregulated by exercise, the additional benefit of supplementation appears to be markedly attenuated [70,76]. Furthermore, the relative efficacy of NR versus NMN in humans remains unresolved, as head-to-head comparisons are lacking and tissue-specific bioavailability differs between compounds [71,77].

### 4.3. Acetyl-L-Carnitine

Acetyl-L-carnitine (ALCAR) regulates acetyl-CoA balance during high-intensity exercise, preventing its accumulation and maintaining pyruvate dehydrogenase (PDH) activity [78]. By regenerating CoASH, it sustains tricarboxylic acid cycle (TCA) flux and fatty acid oxidation while preventing ETC overload [79]. Functionally, ALCAR enhances metabolic flexibility, reduces lactate accumulation, preserves glycogen, and improves tolerance to high-intensity intermittent exercise [80]. These effects, however, are primarily documented in studies using intravenous or high-dose oral administration protocols, which raises questions about practical applicability, given the limited bioavailability of oral ALCAR at standard supplemental doses [78,81]. Moreover, controlled trials in resistance-trained athletes are scarce, and the evidence base is insufficient to make unequivocal recommendations for performance enhancement in this population [81,82].

### 4.4. Amino Acids

Amino acids act as anabolic signals and metabolic substrates. Leucine activates mTORC1, stimulating protein synthesis and mitochondrial protein translation [83,84]. This coordination ensures balanced muscle hypertrophy and oxidative capacity [84]. Branched-chain amino acids (valine, isoleucine) replenish TCA intermediates, especially during glycogen depletion [85,86]. Amino acids also support glutathione synthesis, protecting mitochondrial function and sustaining oxidative phosphorylation [87]. It should be noted, however, that the specific mitochondrial effects attributed to BCAA supplementation are difficult to isolate from the broader effects of adequate total protein intake. In individuals already consuming sufficient dietary protein, additional BCAA supplementation may offer limited incremental benefit for mitochondrial function [83]. The evidence for glutamine’s mitochondrial effects in non-clinical athletic populations similarly remains limited [87,88].

### 4.5. Vitamins and Antioxidants

Vitamin C (ascorbic acid) supports redox balance, endothelial function, and oxygen delivery during exercise, but high doses may impair mitochondrial adaptations by blunting ROS signaling [89,90,91]. Vitamin E protects membrane integrity and mitochondrial function but may also reduce training adaptations when over-supplemented [92,93]. Glutathione (GSH) is essential for redox regulation, calcium handling, and fatigue resistance, though excessive supplementation may impair adaptive signaling [94,95,96]. The blunting effect of high-dose antioxidant supplementation on training-induced mitochondrial adaptations is a critical limitation that is underappreciated in the literature. Several meta-analyses and RCTs have reported that chronic supplementation with vitamins C and E at supraphysiological doses attenuates PGC-1α signaling, reduces AMPK activation, and diminishes long-term gains in VO_2_max and insulin sensitivity in response to endurance training [97,97,98]. This paradoxical effect suggests that physiological ROS generated during exercise serve as essential signaling molecules for mitochondrial adaptation, and indiscriminate scavenging may be counterproductive in healthy trained individuals [98].

Polyphenols enhance endogenous antioxidant defenses, reduce inflammation, and improve recovery and vascular function [99,100]. However, the bioavailability of isolated polyphenol compounds is highly variable and often low, and the translation of in vitro results to meaningful in vivo effects remains a challenge—notably, high-dose resveratrol has also been reported to blunt aerobic training adaptations in some RCTs [98,99].

Coenzyme Q10 supports electron transport and ATP production, reducing fatigue and improving endurance, particularly in individuals with lower mitochondrial function [101,102,103]. The benefit of CoQ10 supplementation appears to be greatest in populations with pre-existing deficiency (e.g., statin users, elderly individuals), and evidence of meaningful ergogenic effects in healthy athletes with replete CoQ10 status is inconsistent [103,104]. Mitochondria-targeted antioxidants such as MitoQ and ergothioneine directly reduce mitochondrial ROS, preserving enzyme function and bioenergetics [10,39,105]. MitoQ supplementation has been shown to reduce oxidative damage and improve performance in cyclists [104,106], while ergothioneine enhances antioxidant defenses and ATP production [107,108]. Unlike general antioxidants, they do not appear to blunt mitochondrial adaptations [104,109]. Nevertheless, the evidence base for both compounds in humans remains limited. In this sense, the study on the effects of MitoQ in cyclists was conducted with a small sample size and should be considered preliminary [109], whereas ergothioneine has been studied almost exclusively in vitro and in animal models, with virtually no RCT data on physical performance in humans [107]. Thus, extrapolating these findings to practical supplementation recommendations is therefore premature.

### 4.6. Urolithin A

Urolithin A (UA), derived from dietary polyphenols via gut microbiota, promotes mitophagy through PINK1/Parkin pathways and regulates mitochondrial dynamics [110,111]. It also stimulates mitochondrial biogenesis via PGC-1α, improving oxidative capacity and ATP production [110]. Animal and human studies show improved mitochondrial function, muscle strength, and fatigue resistance with supplementation [15,112,113]. UA also exerts anti-inflammatory effects by modulating NF-κB and cGAS/STING signaling, reducing cytokines such as TNF-α, IL-1, and IL-6 [36,114,115,116]. However, a critical constraint on urolithin A’s applicability is the marked interindividual variability in its endogenous production: only approximately 40% of the population harbors the gut microbiota capable of converting ellagitannins into UA, rendering food-based strategies ineffective for a large proportion of individuals [117]. Although direct supplementation with pure UA circumvents this limitation, clinical trials conducted to date have primary involved sedentary older adults, and it remains unclear whether the observed mitochondrial and functional benefits extend to younger, trained populations, in whom exercise already ensures effective maintenance of mitochondrial quality control [15,112,117]. The anti-inflammatory effects, though mechanistically plausible, similarly require validation in exercise-specific contexts before clinical recommendations can be made.

## 5. Mitochondrial Adaptations: From Physical Enhancement to Metal Disorders

Mitochondria are key organelles for energy production, redox regulation, and cellular signaling. Both the brain and skeletal muscle have high energetic demands, yet their mitochondrial adaptations differ depending on whether the context is physiological—moderate exercise and a balanced diet—or pathological—chronic stress, very intense exercise, and extreme diets.

### 5.1. Physical Enhancement

In skeletal muscle, PGC-1α is activated during physical exercise through energy and redox sensors such as AMPK and the NAD^+^-dependent deacetylase sirtuin 1 (SIRT1), promoting the synthesis of new mitochondria, increasing oxidative capacity, and optimizing ATP production [114]. This activation can enhance the efficiency of oxidative phosphorylation, regulate ROS accumulation, and support muscle regeneration, fatigue resistance, and post-exercise recovery [118]. During muscle contraction and high-intensity training, transiently generated ROS act as adaptive signals that induce mitochondrial biogenesis and angiogenesis, enhancing the activation of PGC-1α and transcription factors such as NRF-1 and NRF-2 [119]. This increases mitochondrial density, optimizes oxygen and nutrient delivery, and improves metabolic efficiency in muscle, key aspects for elite athlete performance. Maintaining an appropriate ROS balance is essential: controlled levels act as adaptation and survival signals, whereas excessive ROS can cause oxidative damage, inflammation, and delayed muscle recovery. In elite athletes, the coordinated activation of PGC-1α, mitophagy, and redox balance regulation integrates muscular and neuronal adaptations [120].

Mitochondrial biogenesis, mitophagy, and oxidative efficiency are synergistically enhanced, enabling athletes to sustain prolonged efforts, recover faster, improve fatigue resistance, and maintain focus and cognitive function during high-intensity training and competition [121]. These adaptations are modulated differently depending on muscle fiber type, which is critical for performance. Type I fibers, characterized by slow contraction and high endurance, have high mitochondrial density and a predominantly oxidative profile [122]. In these fibers, PGC-1α activation and controlled ROS signaling during endurance training enhance mitochondrial biogenesis, increase energy efficiency, and improve oxygen delivery, prolonging performance in long-duration sports such as marathon running or road cycling [120]. Type II fibers, characterized by fast contraction and high power, have lower mitochondrial density and rely predominantly on anaerobic metabolism. During high-intensity training, PGC-1α activation, mitophagy, and ROS signaling improve oxidative efficiency, facilitate ATP regeneration, and ensure rapid recovery between efforts, which is essential in explosive sports such as sprinting, Olympic weightlifting, or team sports [123]. The integration of these adaptive responses between type I and II fibers allows athletes to optimize both endurance and power, maximizing the overall energetic efficiency of the muscle [124].

However, it is important to note that these adaptations are beneficial only when the training stimulus occurs within the context of adequate energy availability and recovery. When the volume or intensity of training exceeds the organism’s capacity for recovery, overtraining syndrome (OTS) may develop. This condition, which has been documented predominantly in elite athletes and military personnel, leads to biological consequences paradoxically similar to those observed in mitochondrial dysfunction associated with psychiatric disorders [125]. The excess of ROS generated during disproportionate training loads surpasses the cellular antioxidant capacity, leading to lipid, protein, and mitochondrial DNA peroxidation. A reduction in oxidative phosphorylation has also been documented due to impaired coupling between mitochondrial adenylate kinase (AK2) and the adenine nucleotide translocator (ANT), resulting in a decline in the overall ADP-dependent ATP synthesis capacity [28]. In parallel, markers of disrupted redox homeostasis have been consistently identified in athletes with OTS, including increased total oxidative capacity and reduced levels of antioxidants such as erythrocyte glutathione, coenzyme Q10, and carotenoids [126]. These overtraining-induced mitochondrial alterations show a striking molecular similarity to those described in major depression and bipolar disorder, in which ROS accumulation in the absence of an adequate antioxidant response leads to persistent oxidative stress, neuroinflammation, and impaired synaptic plasticity [19]. Thus, the relationship between exercise and mitochondria is neither simply linear nor unidirectional. In this regard, moderate and well-dosed training activates protective adaptive programs, whereas chronic overtraining without sufficient recovery can reverse these benefits and cause the athlete’s mitochondrial phenotype to shift toward a pathological state, highlighting that intensity, frequency and nutritional context are critical determinants of the biological outcome [28,126].

### 5.2. Mental Adaptation

Although mitochondrial plasticity is less pronounced in the brain than in skeletal muscle, regular exercise stimulates mitochondrial biogenesis in regions such as the hippocampus, enhancing antioxidant capacity and promoting neurogenesis [122]. Moreover, exercise further modulates systemic and central inflammation, indirectly optimizing neuronal mitochondrial function. These adaptations are associated with improved cognitive performance, emotional regulation, and stress resilience. In this sense, PGC-1α plays a crucial role in the central nervous system, regulating energy metabolism, mitochondrial biogenesis, and antioxidant defense [127]. In neurons, PGC-1α modulates the expression of antioxidant enzymes and detoxifies ROS while regulating the expression of BDNF, a neurotrophic factor critical for synaptic plasticity, the formation of new neuronal connections, and cognitive and emotional resilience [128]. In glial cells, PGC-1α and mitophagy help control the inflammatory response and protect against oxidative stress by regulating microglial and astrocyte activation and maintaining cerebral homeostasis [129]. Dietary patterns also exert a profound influence on these processes. Chronic hypercaloric diets, particularly those rich in saturated fats and refined sugars, seem to promote neuroinflammation, impair mitochondrial biogenesis, and reduce hippocampal BDNF expression, contributing to deficits in memory, executive function, and emotional regulation [130,131]. Conversely, dietary supplementation with omega-3 polyunsaturated fatty acids (PUFAs), especially DHA and EPA, supports the integrity of neuronal membrane, enhances synaptic plasticity, and boosts PGC-1α and BDNF signaling pathways, thereby improving cognitive flexibility and emotional resilience [132,133]. Furthermore, ketogenic and caloric restriction dietary strategies seem to activate mitochondrial biogenesis and mitophagy in the brain, reducing oxidative stress and attenuating neuroinflammatory cascades implicated in mood disorders and cognitive decline [134]. Polyphenol-rich diets, including those adhering to the Mediterranean dietary pattern, similarly modulate microglial activation and astrocyte function, reinforcing cerebral homeostasis and offering neuroprotective effects relevant to the prevention of neurodegenerative and neuropsychiatric conditions [135,136].

A key molecular bridge connecting skeletal muscle adaptation in athletes with brain function is the PGC-1α/FNDC5/irisin pathway. During exercise, the activation of PGC-1α in muscle stimulates the expression of FNDC5, whose cleaved form, irisin, is secreted into the circulation, crosses the blood–brain barrier, and induces hippocampal BDNF expression along with a broader neuroprotective gene program [137,138]. Circulating irisin levels positively correlate with hippocampal BDNF in a dose-dependent manner, with an intensity threshold around 50% of maximal aerobic velocity, highlighting that the neuroprotective effects of exercise depend not only on its practice but also on its intensity [139]. In athletes, this muscle–brain crosstalk is systematically activated, sustaining hippocampal neurogenesis, synaptic plasticity, and emotional resilience. In contrast, in individuals with depression and other psychiatric disorders, reduced physical activity, mitochondrial dysfunction, and impaired PGC-1α signaling collectively attenuate irisin secretion, contributing to lower hippocampal BDNF levels and to the neurobiological substrate of cognitive and affective symptoms [9,140]. This contrast demonstrates that the neuroprotective effects of exercise are not merely secondary to cardiovascular fitness but are mechanistically rooted in the adaptive mitochondrial capacity of skeletal muscle itself. However, in this context as well, excess can compromise these benefits. In relative energy deficiency in sport (REDs), defined by the International Olympic Committee as a syndrome caused by prolonged low energy availability in athletes of both sexes [141], reduced T3 due to caloric restriction impairs mitochondrial ATP production from glycogen and limits phosphocreatine regeneration [142]. This negatively affects physical performance as well as cognitive function, mood, and mental health, with mechanistic overlap in the psychiatric disorders discussed in this section.

### 5.3. Mental Disorders

Although elite sport is generally associated with health benefits, epidemiological data reveal a paradox: the prevalence of mental disorders among high-performance athletes is not lower than in the general population and, in several categories, it is higher. Meta-analyses of active and retired elite athletes indicate that anxiety and depression affect between 19% and 34% of active competitors, and between 16% and 26% of retired athletes, figures that, in general, appear to exceed estimates for the general population [143,144]. Eating disorders, particularly anorexia nervosa and subclinical restrictive behaviors, are especially prevalent in aesthetic, endurance, and weight-class sports, with reported rates ranging from 6% to 45% in female athletes and up to 19% in male athletes depending on the sport category and assessment method, with the highest burden documented in gymnastics, long-distance running, rowing, and figure skating [145]. Moreover, the chronic physical and psychological stress inherent to high-level competition, combined with frequent musculoskeletal injuries, career transitions, and relative energy deficiency, creates a biological environment that shares pathophysiological features with the psychiatric conditions described below, notably mitochondrial dysfunction.

Several mental disorders are associated with mitochondrial alterations in both the brain and skeletal muscle. Adequate redox balance and efficient mitophagy seem to prevent ROS accumulation, neuroglial inflammation, and synaptic plasticity deterioration, factors associated with mental fatigue and cognitive deficits observed in psychiatric disorders such as depression and bipolar disorder [128]. However, ROS accumulation without adequate antioxidant response leads to persistent oxidative stress, neuroglial inflammation, and impaired synaptic plasticity [146]. Patients with major depressive disorder show reduced activity of respiratory chain complexes, decreased ATP production, and increased oxidative stress [146]. In skeletal muscle, individuals with mental disorders also exhibit signs of mitochondrial dysfunction, including reduced oxidative capacity, early fatigue, and metabolic alterations [147]. Reduced PGC-1α expression and mitochondrial density have been observed in the prefrontal cortex and hippocampus, associated with lower ATP availability, neuronal fatigue, and greater susceptibility to oxidative damage [148]. Similarly, in bipolar disorder, redox signaling imbalances and decreased PGC-1α contribute to mitochondrial dysfunction and altered cerebral energy homeostasis [149]. Moreover, not only in bipolar disorder but also in schizophrenia, abnormalities in mitochondrial DNA, impaired oxidative phosphorylation, and dysregulated calcium signaling have been reported, potentially affecting neurotransmission and synaptic plasticity [149]. In this context, Papageorgiou and Filiou (2024) have reviewed how perturbations in mitochondrial dynamics—including fission, fusion, biogenesis, and mitophagy—constitute a transversal mechanistic thread across major psychiatric disorders such as anxiety, depression, bipolar disorder, and schizophrenia, proposing mitochondrial quality control as a critical and underexplored therapeutic target in psychiatry [150]. Some of these changes may be related to sedentary lifestyle, chronic low-grade inflammation, or adverse effects of psychotropic medications [103]. However, growing evidence suggests that systemic mitochondrial dysfunction may constitute an intrinsic component of the biological phenotype of certain psychiatric conditions.

Aerobic exercise has been proposed as a non-pharmacological intervention capable of partially reversing the mitochondrial deficits observed in psychiatric disorders. In patients with schizophrenia, aerobic exercise programs have shown improvements in cognitive function and clinical symptoms, consistent with the overexpression of BDNF and a reduction in neuroinflammation [151]. In bipolar disorder, physical activity has been associated with improvements in mitochondrial aerobic capacity during remission phases, and a longitudinal study specifically documented the recovery of mitochondrial function indices following clinical stabilization [152]. These observations draw a direct parallel with the "athlete model": whereas training systematically builds mitochondrial resilience, psychiatric illness erodes it, and targeted exercise can partially restore it. The magnitude of this reversal, however, remains significantly below the gains observed in healthy trained individuals, reflecting the persistence of intrinsic biological vulnerabilities in psychiatric populations [153]. It should also be noted that exercise prescription in these patients must consider the risk of drifting toward patterns of compulsive exercise or overtraining, especially in contexts of comorbidity with eating disorders. In such cases, excessive exercise in a state of energy restriction may act as an aggravating factor for mitochondrial dysfunction rather than reversing it [154].

The relationship between nutrition, mitochondrial function, and psychiatric disorders extends beyond the classical mood disorder spectrum to encompass eating disorders and metabolic disease. In anorexia nervosa (AN), severe and prolonged caloric restriction directly impairs mitochondrial bioenergetics. An inhibition of complex I activity and reduced mitochondrial membrane potential have been found in leukocytes of anorexic patients, alongside systemic oxidative stress and depleted glutathione pools [155,156]. Animal models of activity-based anorexia further show that cortical mitochondrial fission is significantly increased at maximum weight loss, a disruption that appears to be partially reversible with weight restoration, although the long-term consequences of transient mitochondrial fragmentation on neuronal connectivity and behavior remain unknown [154]. AN carries the highest mortality rate of any psychiatric illness, and these mitochondrial findings underscore the biological severity of nutritional deprivation on cerebral energy homeostasis [157]. In bulimia nervosa, the cyclical pattern of restriction and binge–purge episodes is associated with chronic oxidative stress, electrolyte imbalances that compromise mitochondrial membrane potential, and impaired cellular redox buffering, although direct mitochondrial studies in bulimic patients are comparatively scarce and constitute an important gap in the literature [158].

From an athletic perspective, the contrast with AN is particularly striking, but it also serves as a relevant warning regarding certain high-risk sporting profiles. While caloric adequacy combined with training enhances mitochondrial biogenesis and oxidative capacity in the skeletal muscle of athletes, severe caloric restriction in patients with AN produces the opposite effect. In animal models, the contribution of complex I to maximum mitochondrial electron transfer is lower than in healthy controls, with impaired muscle performance that persists even after short-term weight recovery [158]. This biological convergence carries a double-edged implication in the context of elite sports: athletes in weight-sensitive disciplines—such as artistic gymnastics, rowing, wrestling, and long-distance running—may develop eating disorders. In these cases, involuntary or deliberate caloric restriction produces a profile of mitochondrial dysfunction, hormonal alteration, and mood deterioration that partially overlaps with that of AN [141,142]. Furthermore, the paradoxical hyperactivity characteristic of many patients with AN does not produce the adaptive mitochondrial responses observed in athletes. In the absence of an adequate energy substrate, exercise in AN, like overtraining without sufficient recovery, accelerates mitochondrial fragmentation instead of promoting biogenesis, acting as a pathological factor rather than a protective one [155,158].

The metabolic–psychiatric interface is further illustrated by the well-established comorbidity between type 2 diabetes (T2DM) and psychiatric disorders. Mitochondrial dysfunction is a cardinal feature of T2DM in both skeletal muscle and the brain: downregulation of PGC-1α and its target genes in insulin-resistant skeletal muscle leads to reduced oxidative phosphorylation efficiency, increased ROS generation, and impaired fatty acid oxidation [159]. In the brain, insulin resistance disrupts neuronal energy metabolism and mitochondrial dynamics, contributing to cognitive decline, depressive symptoms, and increased vulnerability to neurodegenerative processes [160]. This metabolic–neuropsychiatric overlap has led some researchers to propose that T2DM and serious mental disorders share common pathophysiological roots in impaired cellular bioenergetics, a hypothesis consistent with the observation that families with high rates of T2DM exhibit increased prevalence of bipolar disorder and schizophrenia [151]. From an athletic standpoint, T2DM is increasingly documented in former professional athletes—particularly in high-contact and high-body-mass sports such as American football and rugby—where post-career physical inactivity combined with years of caloric surplus create the metabolic substrate for insulin resistance and subsequent metabolic syndrome [161,162]. Nutritional strategies that restore mitochondrial function, including caloric restriction, omega-3 PUFA supplementation, and time-restricted eating, have therefore garnered interest as adjunctive approaches in both metabolic and psychiatric rehabilitation [163]. Critically, T2DM is not only a psychiatric comorbidity in its own right, but also one of the most consistently identified modifiable risk factors for neurodegenerative disease. Chronic hyperglycemia, insulin resistance, and the associated mitochondrial dysfunction create a pathological milieu that accelerates amyloid-β accumulation, tau hyperphosphorylation, and neuronal bioenergetic failure, which are the characteristic molecular features of Alzheimer’s disease (AD) [164]. Meta-analyses of prospective cohort studies estimate that individuals with T2DM face a 56–73% greater risk of developing AD compared to metabolically healthy counterparts, a figure that increases markedly with earlier age of onset and longer disease duration [165]. This relationship is mechanistically so robust that the brain-specific insulin resistance observed in AD has been proposed under the term "type 3 diabetes," reflecting the continuum between peripheral metabolic dysfunction and central neurodegeneration [166,167]. In the athletic context, sports associated with repetitive head trauma—most notably American football, boxing, rugby, and ice hockey—carry additional neurodegenerative risk through traumatic axonal injury and chronic neuroinflammation, which may accelerate the same mitochondrial cascades implicated in AD and chronic traumatic encephalopathy [168].

In AD, cerebral glucose hypometabolism precedes and predicts the accumulation of amyloid-β plaques and tau tangles, implicating bioenergetic failure as an early driver rather than a late consequence of neurodegeneration [169]. Impaired complex I and IV activity, mtDNA damage, reduced glycolytic and oxidative enzymes, and dysregulated mitophagy converge to reduce neuronal ATP supply and amplify oxidative stress, accelerating synaptic loss and cognitive decline [170]. In this context, nutritional interventions targeting mitochondrial metabolism have attracted considerable scientific attention. The ketogenic diet (KD) has been proposed to bypasses the glucose metabolic defect in AD neurons by shifting cerebral fuel utilization from glucose to ketone bodies. Indeed, KD seems to reduce oxidative stress, attenuate neuroinflammation, stimulate mitochondrial biogenesis via PGC-1α, and enhance neuronal autophagy and BDNF expression in both animal models and early clinical trials [170,171]. Similarly, Omega-3 polyunsaturated fatty acids (DHA/EPA) appear to preserve mitochondrial membrane integrity, reduce amyloid burden, and support synaptic plasticity in AD models [172]. However, translational evidence from large-scale RCTs remains limited, and the efficacy of these dietary strategies in established AD, as opposed to early or prodromal stages, requires further investigation before definitive clinical recommendations can be made [170].

Parkinson’s disease (PD) shares with AD a core mitochondrial pathophysiology, though its primary locus of dysfunction lies at complex I of the electron transport chain, where impaired activity leads to dopaminergic neuronal loss in the substantia nigra [173]. The pathological form of α-synuclein protein, whose aggregation into Lewy bodies defines PD, directly interacts with mitochondrial membranes, impairing their function and triggering defective mitophagy via disruption of the PINK1/Parkin pathway [11,174]. Mutations in PINK1 and Parkin genes are among the most common causes of familial PD, underscoring that the mechanism of mitophagy, which is essential for athletic adaptation, is precisely one of the systems whose failure drives neurodegeneration [11]. Epidemiological evidence suggests a 20–30% reduction in the risk of developing PD associated with higher levels of moderate to vigorous physical activity [175]. Furthermore, animal models and preliminary clinical data indicate that aerobic exercise upregulates mitochondrial biogenesis, activates PINK1/Parkin-dependent mitophagy, reduces α-synuclein aggregation, and preserves dopaminergic neuron integrity [175,176].

**Figure 4:**
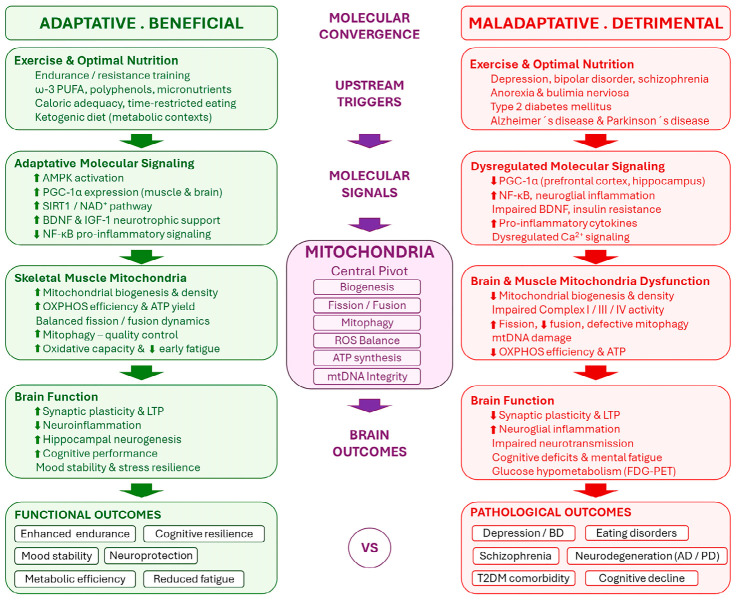
**Mitochondrial adaptations in athletes versus mitochondrial dysfunction in psychiatric and metabolic disorders: a shared axis in opposite directions.** The diagram illustrates the parallel but opposing trajectories of mitochondrial regulation in the context of exercise/optimal nutrition (left, green) and psychiatric, metabolic, and neurodegenerative conditions (right, red). Upward arrows indicate an increase, whereas downward arrows indicate a decrease.

Thus, the mitochondrial adaptations that occur in athletes due to both exercise and nutrition are quite similar to those observed in mental disorders, although they occur in the opposite direction (Figure 4). While in athletes, adaptations are functional and enhance energetic efficiency and cellular resilience, in mental disorders, dysregulation predominates, leading to reduced bioenergetic capacity and increased oxidative stress [149]. Moreover, whereas mitochondrial adaptations in skeletal muscle in response to exercise are robust and readily quantifiable, those in the brain are generally more subtle and strongly modulated by neurotrophic and inflammatory factors [123]. Collectively, these observations highlight the metabolic interplay between brain and muscle and support the notion that interventions such as exercise or nutrition may beneficially modulate mitochondrial function in both contexts, representing a promising but still insufficiently explored therapeutic frontier that spans psychiatry, metabolic medicine, and neurology.

## 6. Future Perspectives

This study suggests a transition toward highly personalized training and nutritional strategies, grounded in a detailed analysis of each athlete’s individual metabolic profile. It is a priority to further investigate the role of mitochondria, not only as the muscular energy powerhouse but also as a key regulator of cognitive and emotional resilience. This includes exploring the “lactate–brain axis” and the activation of the SIRT1–PGC-1α pathway to mitigate mental disorders derived from athletes’ interventions. Furthermore, the use of specific modulators and antioxidants opens new avenues for optimizing performance and recovery without interfering with the body’s adaptive signaling, overcoming the limitations of conventional supplements. Ultimately, integrating these metabolic interventions holds the potential to transform elite physical preparation and recovery, alongside the therapeutic management of neuropsychiatric disorders (Table 1).

## Figures and Tables

**Table 1:** Strengths, weaknesses, opportunities, and challenges of mitochondrial adaptations.

Strengths (Endogenous and Adaptative Mechanisms)	Opportunities (Supplementation Line Therapeutic Targets)
Efficiency of PCr shuttle: High capacity for ATP resynthesis via mitochondrial creatine kinase, optimizing energy transfer between sites of production consumption.	Modulation via NAD+ precursors: NMN and NR restore NAD+/NADH balance, activating SIRT1 and promoting mitochondrial biogenesis even in fatigue.
Mitochondrial plasticity: Upregulation of mitochondrial biogenesis through the activation of AMPK and transcriptional coactivator PGC-1α.	Metabolite-driven activation of cellular clearance promotes removal of dysfunctional mitochondria, enhancing muscle strength and aerobic endurance.
Lactate clearance capacity: High expression of MCT1 and MCT4 enabling efficient lactate transport for oxidation in type I fibers and gluconeogenesis.	Mitochondria-targeted antioxidants: Molecules such as MitoQ accumulate in the inner mitochondrial membrane, neutralizing hydrogen peroxide without disrupting cytosolic redox signaling.
Robustness of quality control systems: Efficient activation of PINK1/Parkin-dependent mitophagy, ensuring a functional mitochondrial population and reducing electron leakage.	Kinetic optimization of creatine: Creatine improves mitochondrial ADP sensitivity, accelerating steady-state transition and reducing the initial oxygen deficit.
Adaptive redox homeostasis: Cellular and muscle fiber capacity to manage transient increases in ROS as signaling molecules for hypertrophy and angiogenesis.	Lactate–brain axis: Leveraging lactate as cerebral fuel and using supplements that enhance BDNF expression, connecting physical performance with mental health and cognitive sharpness.
**Weaknesses (Biochemical and Kinetic Limitations)**	**Threats (Interference and Risks)**
Anaerobic glycolysis kinetics: The drop of intracellular pH due to the accumulation of H+ inhibits PFK-1, limiting glycolytic flux.	Hormetic attenuation by antioxidants: Chronic use of high doses of antioxidants may blunt the signaling required for the expression of GLUT4 and oxidative enzymes.
ECT saturations: A massive increase in energy demand can exceed the capacity of complexes I–IV, leading to increased superoxide production and oxidative stress.	Metabolic disruption from diets: Loss of metabolic flexibility to switch between using fat and carbohydrates due to poorly periodized nutritional protocols.
Glycogen store depletion: Dependence on limited muscle and hepatic glycogen stores, with their depletion compromising the ability to maintain exercise intensity above the critical power threshold.	Overtraining and MQC dysfunction: The risk that chronic mechanical stress exceeds cellular repair capacity, leading to the accumulation of damaged mitochondria and persistent fatigue.

## Data Availability

The original contributions presented in this study are included in the article/Supplementary Materials. Further inquiries can be directed to the corresponding author.

## Supplementary Materials

The following supporting information can be downloaded at: https://www.mdpi.com/article/10.3390/cimb48050498/s1. References [59,60,61,62,63,64,65,66,67,68,69,70,71,72,73,74,75,76,77,78,79,80,81,82,83,84,85,86,87,88,89,90,91,92,93,94,95,96,97,97,98,99,100,101,102,103,104,105,106,107,108,109,110,111,112,113,114,115,116,117] are cited in the Supplementary Materials.
