# Supplementary material for: Revision of Energy Metabolism Adaptations in High-Level Athletes: From Physical Performance Enhancement to Potential Therapeutic Targets in Mental Disorders
Source: Curr Issues Mol Biol. 2026 May 11;48(5):498. doi: 10.3390/cimb48050498 (PMC13204626; doi:10.3390/cimb48050498)
Supplement: Supplementary file 1 [file cimb-48-00498-s001.zip › cimb-4255068-supplementary.pdf]

Supplementary Table S1. Nutritional modulators of mitochondrial function and exercise adaptations: evidence level, effect magnitude, and limitations.

Supplement	Mitochondrial Effect	Exercise Adaptations	Main Mechanism	Level of Evidence	Magnitude of Effect	Limitations	Ref.
**Creatine**	↑ Mitochondrial biogenesis; ↑ ADP sensitivity of respiration; ↑ redox buffering	↑ VO_2max; ↑ oxidative phosphorylation efficiency; ↑ fatigue resistance; ↓ ROS damage; improved recovery	AMPK → PGC-1α → NRF-1/NRF-2/TFAM; ↑ mtCK-mediated ADP flux; ↑ NAD^+/NADH ratio; ↑ NADPH and GSH regeneration	In vitro and In vivo and Multiple RCTs (high)	Moderate–high: ↑ strength 5–15%, ↑ lean mass 1–2 kg; modest VO_2max gains (~3–5%)	Responder variability (non-responders ~25–30%); GI discomfort at loading doses; limited long-term data on mitochondrial biogenesis endpoints in humans; most RCTs short-term (≤12 wk)	[59–68]
**NAD^+ Precursors (NR/NMN)**	↑ NAD^+ availability; ↑ Complex I flux; ↑ mitochondrial protein deacetylation; ↑ mitophagy	↑ Functional VO_2max; ↑ submaximal oxidative capacity; improved mitochondrial turnover	NAMPT salvage pathway; SIRT1–PGC-1α activation; SIRT3-mediated deacetylation (IDH2, SOD, Complex I); PINK1/Parkin pathway	Robust in vitro and In vivo (rodents); emerging clinical evidence (small RCTs)	Moderate in older/sedentary adults (~4–13% ↑ VO_2max); minimal in trained individuals	Most human trials small (n < 50) and short-term; tissue-specific bioavailability uncertain; optimal dose and form (NR vs. NMN) not established; effects in trained athletes unclear	[69–77]
**Acetyl-L-Carnitine**	Maintains acetyl-CoA/CoASH balance; sustains TCA flux; prevents ETC reductive overload	↓ Lactate accumulation; delayed ventilatory threshold; ↑ metabolic flexibility; ↑ intermittent performance	CAT buffering of acetyl groups; preservation of PDH activity; support of β-oxidation and oxidative metabolism	In vitro and In vivo and Some clinical trials (moderate)	Moderate: ↓ lactate 10–20% at submaximal loads; modest improvements in time-to-exhaustion	Bioavailability varies with oral dosing; limited RCTs in healthy athletes; GI side effects at high doses; no established optimal loading protocol; evidence for mitochondrial biogenesis endpoints in humans is weak	[78–82]
**Amino Acids (EAA/BCAA/Glutamine)**	Coordination of mitochondrial expansion with hypertrophy; TCA anaplerosis; support of GSH pool	↑ Myofibrillar protein synthesis; maintenance of oxidative capacity; improved metabolic stability during glycogen depletion	mTORC1 activation; anaplerotic conversion to succinyl-CoA; nitrogen donation for GSH synthesis	In vitro and In vivo and Multiple RCTs (high for protein synthesis; moderate for mitochondrial endpoints)	High for muscle protein synthesis (leucine threshold); moderate for metabolic/mitochondrial outcomes	Mitochondrial-specific effects often confounded by total protein intake; diminishing returns with adequate dietary protein; BCAA supplementation may be redundant with sufficient protein; limited evidence on glutamine for mitochondrial	[83–88]

Supplement	Mitochondrial Effect	Exercise Adaptations	Main Mechanism	Level of Evidence	Magnitude of Effect	Limitations	Ref.
						endpoints in non-clinical populations	
**Vitamin C**	Cytosolic ROS scavenging; regeneration of α-tocopherol; modulation of NO bioavailability	Maintains redox balance during endurance; may blunt mitochondrial biogenesis with chronic high doses	Direct ROS scavenging; modulation of PGC-1α and MAPK redox signaling	In vitro and In vivo and Several RCTs (moderate)	Small–moderate protective effect on oxidative stress markers; negative effect on adaptations with high chronic dosing	Paradoxical blunting of training adaptations at doses >1 g/day; optimal dose for performance is unclear; high doses may impair insulin sensitivity; confounding by habitual dietary intake in many trials	[89–91]
**Vitamin E**	Protection of mitochondrial membrane lipids; preservation of membrane protein integrity	Reduced membrane damage in high-intensity/eccentric exercise; excessive doses may blunt oxidative adaptations	Termination of lipid peroxidation chain reactions in mitochondrial membranes	In vitro and In vivo + Some clinical trials (moderate)	Small: modest ↓ lipid peroxidation markers; high doses associated with attenuated aerobic adaptations	Supraphysiological doses blunt mitochondrial biogenesis and AMPK signaling; fat-soluble accumulation risk with chronic high dosing; interactions with anticoagulants; combined with Vitamin C may amplify blunting of adaptations	[92,96]
**Glutathione (GSH)**	Intracellular redox buffering; regulation of H_2O_2 signaling; protection of Fe–S clusters	Sustains force production; delays peripheral fatigue; excessive reinforcement may blunt adaptations	GPx–GR system; modulation of mitochondrial enzyme activity and Ca^{2+} handling	In vitro and In vivo (moderate); limited direct clinical evidence for exogenous GSH	Moderate for indirect precursors (e.g., NAC); direct oral GSH bioavailability is poor	Oral bioavailability of exogenous GSH is very low; NAC (precursor) better studied but also associated with blunting of training adaptations at high doses; dose-response relationships poorly characterized in athletes	[94–99]
**Dietary Polyphenols (Resveratrol, Quercetin, etc.)**	Activation of endogenous antioxidant pathways; improved endothelial function	↓ DOMS; improved recovery; enhanced vascular responses	Nrf2 activation; modulation of inflammatory signaling	In vitro and In vivo and Growing clinical evidence (moderate)	Small–moderate: ↓ inflammatory markers 15–30%; limited direct performance benefits in trained athletes	Low and variable bioavailability (especially resveratrol); matrix-dependent absorption; high doses of resveratrol may blunt aerobic training adaptations; most RCTs use isolated	[99–101]

Supplement	Mitochondrial Effect	Exercise Adaptations	Main Mechanism	Level of Evidence	Magnitude of Effect	Limitations	Ref.
						compounds rather than food-derived polyphenols; translation from in vitro results is often limited	
**Coenzyme Q10 (CoQ10)**	Electron carrier in ETC; preservation of mitochondrial membrane integrity	↓ Perceived fatigue; ↑ time-to-exhaustion	Electron transfer between Complexes I–III; ubiquinol antioxidant activity	In vitro and In vivo and Multiple RCTs (moderate)	Small–moderate: ↓ fatigue perception; marginal improvements in VO_2max (~3–4%) mainly in CoQ10-deficient or older individuals	Highly lipophilic — bioavailability varies with formulation; less effective in already-replete healthy adults; expensive; most benefit seen in deficient states (e.g., statin users, elderly); inconsistent results across exercise types	[102–105]
**MitoQ**	Targeted mitochondrial ROS neutralization (Complex I and III)	↓ Lipid peroxidation; ↑ time-trial performance (~1.3%); delayed fatigue; preserved biogenesis signaling	Accumulation in inner mitochondrial membrane via electrochemical gradient; direct ROS scavenging at ETC	In vitro and In vivo (robust); limited human RCTs	Promising but small sample sizes; ~1.3% TT improvement (1 RCT); consistent ↓ oxidative stress markers	Very limited human data (n < 30 in most trials); long-term safety not established; high cost; optimal dosing unclear; unclear whether mitochondrial ROS scavenging also blunts redox-mediated adaptation signaling at high doses	[106–110]
**Ergothioneine**	Mitochondrial ROS scavenging; preservation of oxidative enzymes	Sustained ATP production under high demand; maintained oxidative phosphorylation efficiency	OCTN1-mediated mitochondrial accumulation; ↑ SOD/catalase activity; protection of PDH & ATP synthase	In vitro and In vivo; minimal clinical evidence	Preclinical results promising; human performance data essentially absent	Virtually no human RCTs on exercise performance; dietary sources limited (mushrooms); supplemental form and optimal dose unknown; mechanistic evidence largely from cell culture; evidence level significantly lower than other supplements in this table	[106–110]

Supplement	Mitochondrial Effect	Exercise Adaptations	Main Mechanism	Level of Evidence	Magnitude of Effect	Limitations	Ref.
**Urolithin A**	↑ Mitophagy; ↑ mitochondrial quality and biogenesis; ↑ oxidative capacity; ↑ ATP production; ↑ ROS control	↑ Muscle endurance and strength; ↑ fatigue resistance; improved recovery	PINK1/Parkin-mediated mitophagy; modulation of mitochondrial dynamics; PGC-1α activation; anti-inflammatory via NF-κB and cGAS/STING	In vitro and In vivo and Early-phase RCTs (growing clinical evidence)	Moderate: ↑ muscle endurance ~8–12% (phase IIa RCT); improved mitochondrial gene expression in older adults	Dependent on gut microbiome composition for production from ellagitannins (non-producer phenotype in ~40% of population); direct supplementation bypasses this but is expensive; most RCTs in older/sedentary populations — efficacy in trained athletes unclear; long-term safety data limited	[111–118]

Abbreviations: RCT, randomised controlled trial; ETC, electron transport chain; TCA, tricarboxylic acid cycle; ROS, reactive oxygen species; DOMS, delayed-onset muscle soreness; NAC, N-acetylcysteine; NR, nicotinamide riboside; NMN, nicotinamide mononucleotide; TT, time trial; VO_2max, maximal oxygen uptake. ↑–increase, ↓–decrease.
